# Complete chloroplast genome of *Isoetes sinensis*, an endemic fern in China

**DOI:** 10.1080/23802359.2019.1666687

**Published:** 2019-09-27

**Authors:** YaCong Xie, Hui-Shan Cheng, Yu Chen, Zhong-Jian Liu, Si-Zu Lin

**Affiliations:** a College of Forestry, Fujian Agriculture and Forestry University, Fuzhou, China;; b Key Laboratory of National Forestry and Grassland Administration for Orchid Conservation and Utilization at College of Landscape Architecture, Fujian Agriculture and Forestry University, Fuzhou, China;; c Fujian Colleges and Universities Engineering Research Institute of Conservation and Utilization of Natural Bioresources, College of Forestry, Fujian Agriculture and Forestry University, Fuzhou, China

**Keywords:** Isoetes sinensis, chloroplast genome, phylogeny

## Abstract

*Isoetes sinensis* is the only aquatic pteridophyte in China with high research value of phylogeny. It is in endangered status. A conservation strategy is therefore imperative for this endangered pteridophyte. In the study, the complete chloroplast (cp) genome of *Isoetes sinensis* (Isoetaceae) was assembled and annotated. It is the full length of 145,492 bp, include large single-copy (LSC) region of 91,865 bp, small single-copy (SSC) region of 13,207 bp, and a pair of invert repeats (IR) regions of 27,213 bp. Plastid genome contains 135 genes, 71 protein-coding genes, 36 tRNA genes, and eight rRNA genes. Phylogenetic analysis suggested *I. sinensis* was most closely related to the clade of *I. melanospora*, *I. mattaponica*, *I. graniticola*, *I. engelmannii*, *I. flaccida, I. valida*, and *I. butleri*, with strong support (bootstrap = 100%). The cp genome will contribute to further research and conservation of *I. sinensis*.

*Isoetes sinensis* is a member of the Isoetaceae, only distributes in China, discretely distributed in eastern China; Anhui, Jiangsu, Jiangxi, and Zhejiang provinces are the main habitats (Kang et al. 2005). This fern perennially grows in shallow water, wetland or humid soil with slightly acidic condition (Chen et al. 2004). *Isoetes sinensis* is famous for unique morphological characteristics and important position in the phylogeny of pteridophyte (Chen et al. 2004). Blaming to human interference and habitat loss, size of extant populations of *I. sinensis* has decreased sharply (Ye and Li 2003). This leads to *I. sinensis* is listed as an endangered species by IUCN (2004). Published papers have demonstrated to genetic factors have direct and/or indirect impacts on the viability of endangered species (Frankham et al. 2002). The complete chloroplast genomic data will be useful for conservation and phylogenetic studies of *I. sinensis.*

In this study, we assembled the complete cp genome of *I. sinensis*. Fresh leaf sample of *I. sinensis* was acquired from Fuzhou City (119°23′89.30″E, 26°08′76.41″N), Fujian Province of China, and voucher specimen deposited at Herbarium of College of Forestry, Fujian Agriculture and Forestry University (specimen code FAFU3333). DNA extraction from fresh leaf tissue, with 350 bp randomly interrupted by the Covaris ultrasonic breaker for library construction. The constructed library was sequenced PE150 using Illumina Hiseq Xten platform, approximately 1.63 GB data generated. Illumina data were filtered by script in the cluster (default parameter: -L 5, -p 0.5, -N 0.1). Plastid genome assembled using GetOrganelle pipe-line (https://github.com/Kinggerm/GetOrganelle); it can get the plastid-like reads, and the reads were viewed and edited by Bandage (Wick et al. 2015). Assembled chloroplast genome annotation based on comparison with *I. sinensis* by GENEIOUS R11.15 (Kearse et al. 2012). The annotation result was drawn with the online tool OGDRAW (http://ogdraw.mpimp-golm.mpg.de/) (Lohse et al. 2013).

The complete plastid genome sequence of *I. sinensis* (GenBank accession MN172503) was 145,492 bp in length, contains a large single-copy (LSC) region of 91,865 bp, a small single-copy (SSC) region of 13,207 bp, and a pair of inverted repeats (IR) regions of 27,213 bp. A total of 135 gene species were annotated, including 71 protein-coding (PCG), 36 transfer RNA (tRNA), and eight ribosomal RNA (rRNA) gene species. The complete genome GC content was 38.00%. In order to reveal the phylogenetic position of *I. sinensis*, a phylogeny reconstruction was performed based on 11 complete cp genomes of *Isoetes* (*I. cangae, I. serracarajensis, I. nuttallii, I. sinensis, I. melanospora, I. graniticola, I. mattaponica, I. engelmannii, I. butleri, I. valida* and *I. flaccida*) and two taxa (*Alsophila spinulosa* and *Psilotum nudum*) as outgroup, they all downloaded from NCBI GenBank. The sequences were aligned using MAFFT v7.388 (Katoh and Standley 2013), and the maximum-likelihood tree was constructed using XSEDE version 8.2.10 (Stamatakis 2014). The phylogenetic tree demonstrated that *I. serracarajensis* has a close relationship to *I. cangae*, both of them form a branch and lie in the base. *I. nuttallii* is a clade solely. In addition, *I. sinensis* constitutes the third independent branch and is the sister to all other species with 100% bootstrap support (Figure 1).

**Figure 1.**
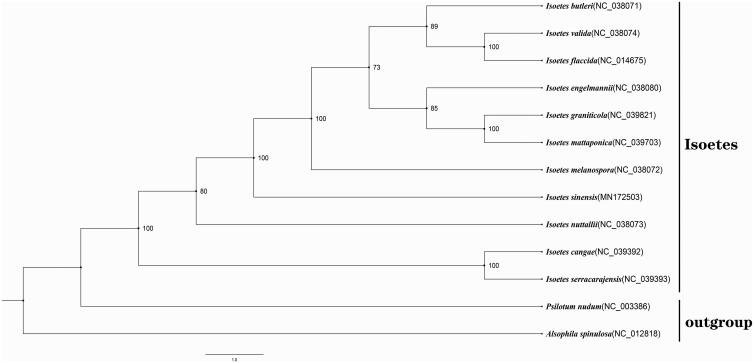
Phylogenetic analysis of 11 species of Isoetaceae and two taxa (*Psilotum nudum*, *Alsophila spinulosa*) as outgroup based on plastid genome sequences by RAxML, bootstrap support value near the branch.
